# Antitumor effect of combination of the inhibitors of two new oncotargets: proton pumps and reverse transcriptase

**DOI:** 10.18632/oncotarget.13792

**Published:** 2016-12-03

**Authors:** Luana Lugini, Ilaria Sciamanna, Cristina Federici, Elisabetta Iessi, Enrico Pierluigi Spugnini, Stefano Fais

**Affiliations:** ^1^ Department of Therapeutic Research and Medicine Evaluation, National Institute of Health, Rome, Italy; ^2^ Department of Servizio Biologico e per la Gestione della Sperimentazione Animale (SBGSA), National Institute of Health, Rome, Italy; ^3^ Stabilimento Allevatore Fornitore Utilizzatore (SAFU) Department, Regina Elena Cancer Institute, Rome, Italy

**Keywords:** proton pump inhibitors, lansoprazole, reverse transcriptase, efavirenz, tumor acidity

## Abstract

Tumor therapy needs new approaches in order to improve efficacy and reduce toxicity of the current treatments. The acidic microenvironment and the expression of high levels of endogenous non-telomerase reverse transcriptase (RT) are common features of malignant tumor cells. The anti-acidic proton pump inhibitor Lansoprazole (LAN) and the non-nucleoside RT inhibitor Efavirenz (EFV) have shown independent antitumor efficacy. LAN has shown to counteract drug tumor resistance. We tested the hypothesis that combination of LAN and EFV may improve the overall antitumor effects. We thus pretreated human metastatic melanoma cells with LAN and then with EFV, both in 2D and 3D spheroid models. We evaluated the treatment effect by proliferation and cell death/apoptosis assays in classical and in pulse administration experiments. The action of EFV was negatively affected by the tumor microenvironmental acidity, and LAN pretreatment overcame the problem. LAN potentiated the cytotoxicity of EFV to melanoma cells and, when administered during the drug interruption period, prevented the replacement of tumor cell growth.

This study supports the implementation of the current therapies with combination of Proton Pumps and Reverse Transcriptase inhibitors.

## INTRODUCTION

Undifferentiated cells and tumor cells often express high levels of endogenous non-telomerase reverse transcriptase of retroposone/retroviral origin [1–3]. Some *in vitro* and *in vivo* studies have shown that this protein plays an important role in the development and progression of several tumors, including melanoma, prostatic, mammary and pancreatic carcinomas [4–6]. Many pharmacological approaches have been attempted to counter this role, including different inhibitors [7]. Our group has been successfully investigating, *in vitro* and *in vivo*, the potential antitumoral action of two inhibitors of the two new oncotargets, Reverse Transcriptase (RT) and Proton Pumps: the non-nucleoside reverse transcriptase inhibitor, Efavirenz (EFV) [4] and the proton pump inhibitor (PPI) Lansoprazole [8, 9] respectively.

EFV is part of the currently adopted multi-agents protocols devised for the treatment of HIV infections [10–12]. Despite its efficacy, EFV is associated with limiting side effects, including neurological disorders, cutaneous eruptions and teratogenesis [13–15]. CNS toxicity, such as dizziness, headaches, and depression and psychiatric events have consistently been reported in 40%–60% and in 25%–40% of patients respectively. This is the main reason for discontinuing/switching therapy [11, 16–18]. Unfortunately, in HIV patients the discontinuing therapy resulted in unresponsiveness upon reinstitution of the protocol. Moreover cases of resistance to EFV have also been emerging [11, 19].

We have shown that the reverse transcriptase inhibitor Efavirenz exert also a potent anti-tumor activity [3–5]. Recently one preclinical study has demonstrated that higher dosage of Efavirenz respect to the 600 mg/day recommended for AIDS treatment, may offer possible benefit for the treatment of metastatic castration-resistant prostate cancer patients [20]. This EFV toxicity due to high dosage used, led us to study a strategy to overcome the EFV toxicity and improved the antitumor response.

Tumor extracellular acidity is one of the key factors responsible for chemoresistance [21, 22]. Two reasons explain the role of pH gradient reversal in chemoresistance, the first one is linked to the protonation of antineoplastic drugs that are weak bases [23] and the second one is linked to the biophysical ultrastructure changes happening at the level of the cellular membrane due to pH changes [24–26]. While the former explains why weak base drugs cannot cross the cell membrane of drug resistant cancer cells, the later provides a solid ground for a similar mechanism for all antineoplastic drugs including weak base. It is therefore a generic concept as far as drug delivery and uptake by cells is involved. Extracellular acidity is associated with cytosolic alkalinity in cancer cells, and this “reverse of pH gradients”, represent a hallmark of malignancy [27, 28]. This microenvironmental acidity is triggered by the lactate accumulation due to the sugar fermentation (Warburg's effect) and the consequent hyperactivity of proton exchangers that pump or exchange the H^+^ outside the cells or inside the internal vacuoles, [22, 29]. This was supported by *in vivo* studies in both animal models and tumor patients [30–32].

We recently developed a novel approach to counter tumor acidity by off-label using PPI [33]. PPI are currently used as anti-acid drugs for treatment of peptic diseases [34, 35]. They are prodrugs which need acidic pH to be transformed in the active molecule (tetracyclic sulfonamide), thus they may accumulate in acidic tissues, activated and act there [36].

Our preclinical investigations have shown that PPI can be used as both chemosensitizers and direct antitumor agents as well [36–42] A wide panel of human tumors have been shown to be responsive to PPI while with different intracellular mechanisms [37, 43, 44] and this might be better managed in tumor patients, by *in vivo* measuring of tumor pH [45].

The pre-clinical studies triggered a series of clinical studies on PPI, in either human patients with cancers and companion animals with spontaneous tumors, supporting the use of these antiacidic compounds in future anti-cancer strategies and excluding any levels of systemic and specific toxicity, even at very high dosage [46–50].

In a recent study we have shown that PPI, while belonging to the same class of generic drugs, have different chemical features, and most of all a different *in vitro* and *in vivo* anti-tumor effect. In fact, Lansoprazole (LAN) was the most effective in terms of cytotoxic effect against a wide panel of human tumor cells and tumors [8]. We have also shown that LAN is very effective in both modulating tumor acidification and enhance sensitivity to suboptimal doses of anti-tumor drugs, such as Paclitaxel, and this was consistent with a significant reduction of systemic toxicity [9].

In this work we demonstrated in 2 and 3D spheroid melanoma models that: 1) LAN enhances the antitumor effect of EFV, 2) the combination of EFV and LAN is more effective than the single agents, 3) LAN can be used in the EFV interruption period, thus preventing the occurrence of tumor chemoresistance towards EFV.

Of interest these two drugs have not been developed as antitumoral agents: Lansoprazole is a well-established first line treatment for gastric hypersecretion and esophageal reflux, while the primary use of EFV is part of multi-drug protocols for the treatment of HIV [11–13]. These results, provide a clear evidence on the use of Proton pumps and Reverse Transcriptase as new oncotargets, and that the combination of specific inhibitors, such as Lansoprazole and Efavirenz, might be considered an innovative and effective new strategy to be implemented to the currently adopted antitumor therapies.

## RESULTS

### Acidic microenvironment of tumor cells reduces Efavirenz efficacy

**Figure 1:**
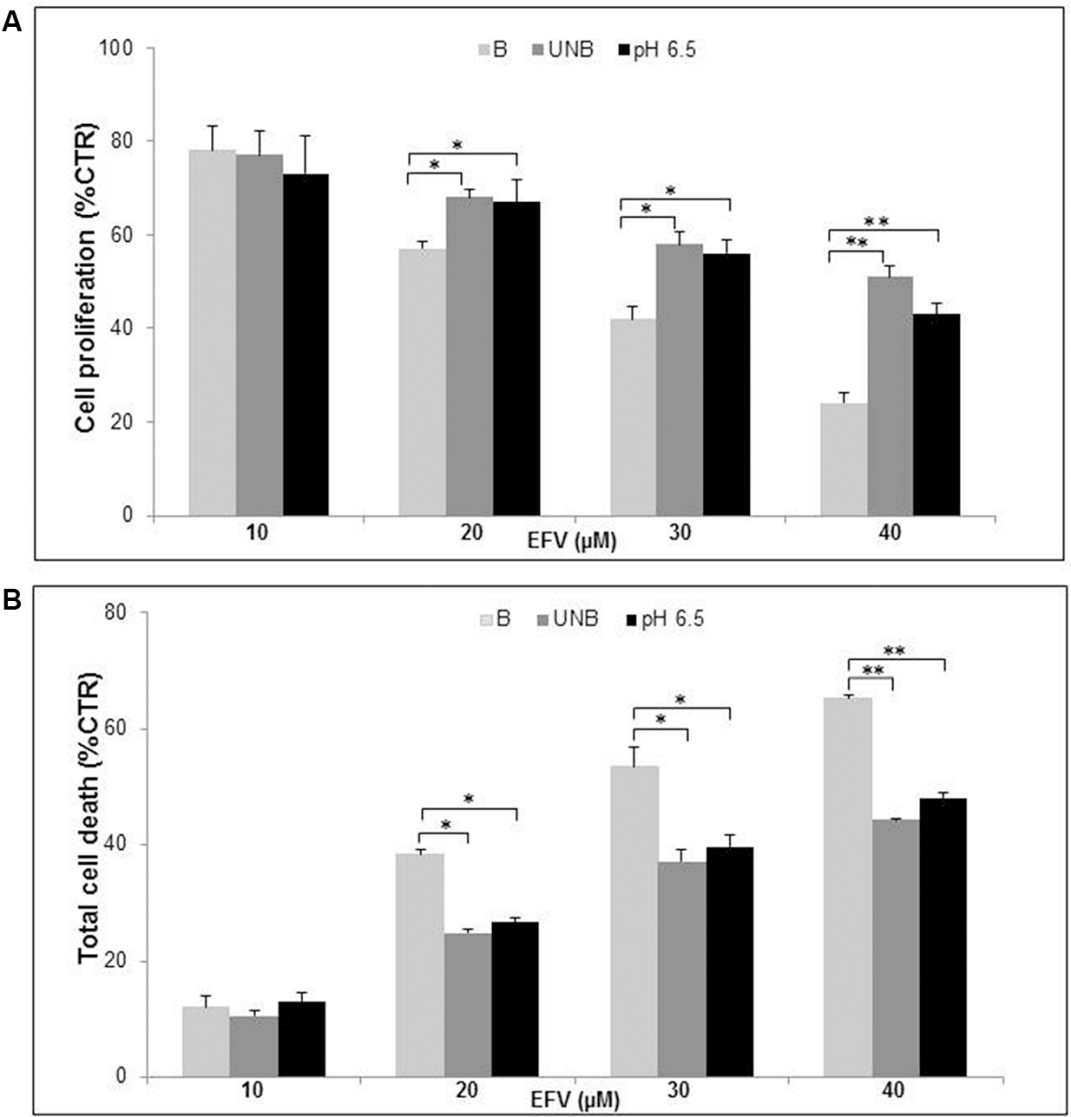
Cell culture pH affected Efavirenz antitumor efficiancy Dose/Response curve of proliferative (**A**) and cytotoxic (**B**) effects of different Efavirenz (EFV) dosage (10, 20, 30, 40 μM) on human Me30966 metastatic melanoma cells treated in buffered (B), unbuffered (UNB) and acidic pH (pH 6.5) conditions for 96 hours. Columns, mean percentages of or cell death of three independent experiments run in triplicate; bars indicate SD. (*) indicate *p* ≤ 0.05and (**) indicate *p* ≤ 0.01.

This set of experiments was aimed at establishing whether the acidic microenvironmental condition could indeed be responsible for some level of low responsiveness of human tumor cells to the action of EFV. To this purpose we initially tested three different conditions for the melanoma cell culture: 1) buffered medium, the cell culture standard condition; 2) unbuffered medium (*in vitro* condition simulating the spontaneous acidification occurring within tumors); 3) pH 6.5 medium (the hypothetical tumor acidic condition, previously used in our melanoma experimental setting) [8]. These two latest conditions, beside mimicking the tumor microenvironment, are also instrumental to allow the activation of PPI, well known prodrugs with a specific delivery for acidic compartments where they are transformed into the active molecule (tetracyclic sulfonamide). In a recent study, we have shown that among several members of the PPI family, Lansoprazole was the one showing the best antitumor efficacy, even at suboptimal doses [8]. We first tested the effect of different pH conditions on the antitumor activity of EFV, in term of either proliferation or cytotoxicity. The results showed that the effectiveness of EFV was much impaired by the acidic conditions. In fact, at EFV concentration of 20, 30 and 40 μM acidic conditions (unbuffered and pH 6.5) highly impaired the EFV inhibitory effect on tumor cell proliferation as compared to the buffered medium (Figure 1A). However, the most clear effect induced by the acidic conditions was on the cytotoxic effect of EFV. These experiments showed that while there was a clear dose dependent cytotoxic effect in the buffered conditions, at unbuffered and acidic pH conditions the percentage of dead cells was always significantly lower (Figure 1B). Based on these results, we established the 20 μM EFV concentration as the reference dose for the following experiments, considering this value as the suboptimal concentration that allowed an adequate cell survival.

### Lansoprazole pre-treatment of melanoma cells enhances Efavirenz anti-tumor effect both in cell monolayer and in 3D spheroid models

**Figure 2:**
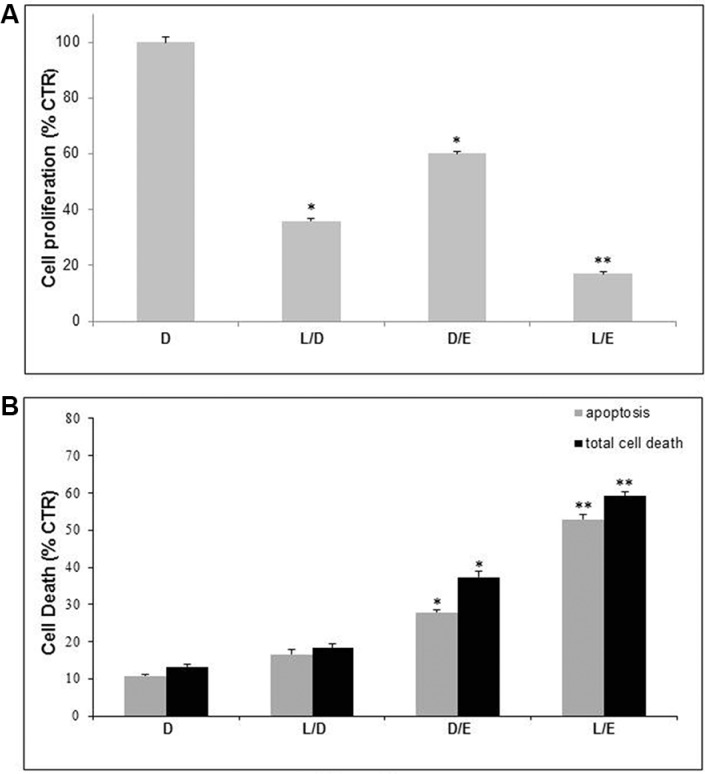
Lanzoprazole pre-treatment affected Efavirenz efficacy on 2D melanoma cell culture Proliferative (**A**) and cytotoxic (**B**) effects of combined treatment of Lansoprazole (L) and Efavirenz (E) on Me30966 melanoma cell monolayer. DMSO (D) as control, L used for 48 hours (50 μM) and E for 96 hours (20 μM). Columns, mean percentages of cell proliferation or cell death of three independent experiments run in triplicate; bars indicate SD. (*) indicate *p* ≤ 0.05 and (**) indicate *p* ≤ 0.01.

This set of experiments was carried out in unbuffered condition, mimicking the acidic microenvironment of tumors [8]. As previously reported, both EFV and LAN had antitumor effect as single agents [3–5, 8, 37, 43]. To explore their potential combinatory effect both drugs were administered at suboptimal concentrations (i.e. 20 μM EFV for 96 hours and 50 μM LAN for 48 hours in monolayer experiments; 30 μM EFV and 75 μM LAN in experiments performed with 3D spheroids). In the experiments performed with standard cell culture conditions (monolayer), the combination of the two drugs led to a significant reduction of cell proliferation (80%) (Figure 2A), as compared to the effect of the single agents. In all conditions the anti-proliferative effect was associated to a cytotoxic effect, again more evident when the two drugs were used in combination, and it was mostly due to apoptosis (Figure 2B).

**Figure 3:**
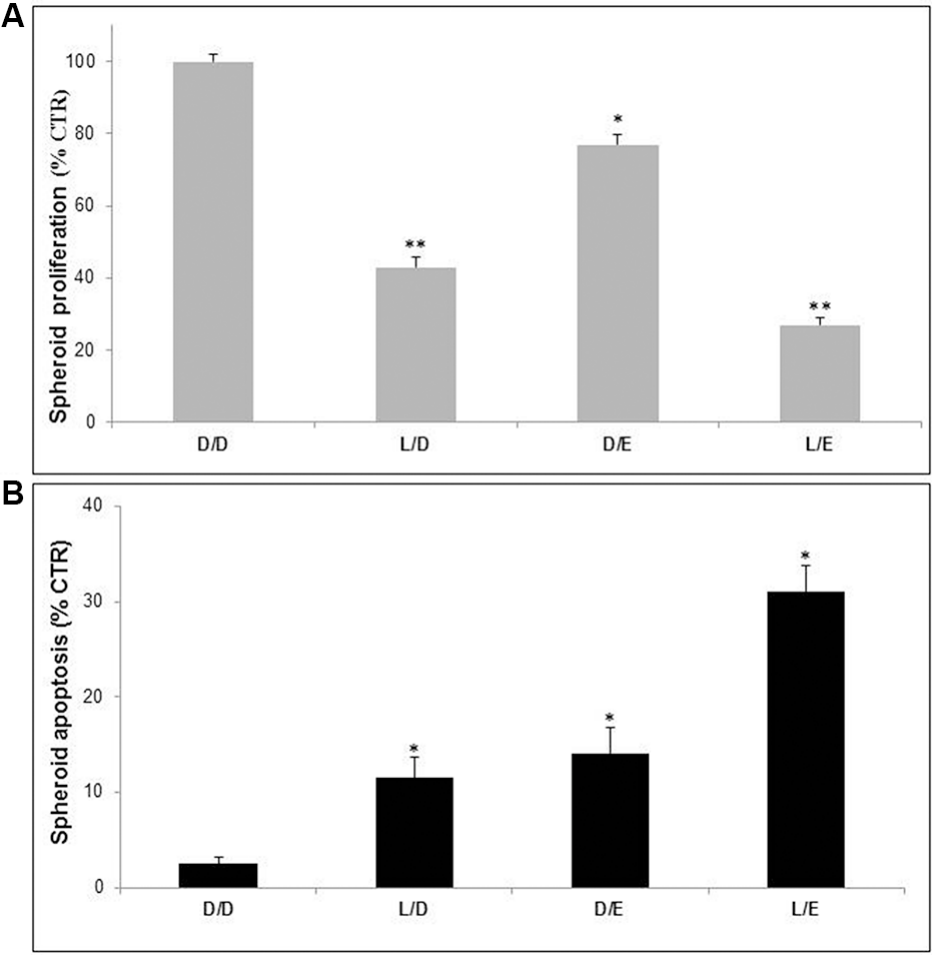
Lanzoprazole pre-treatment affected Efavirenz efficacy on 3D melanoma spheroids Proliferative (**A**) and cytotoxic (**B**) effects of combined treatment of Lansoprazole (L) and EFV (E) on Me30966 melanoma spheroids. DMSO (D) as control, L used for 48 hours (75 μM) and E for 96 hours (30 μM). Columns, mean percentages of cell proliferation and apoptosis of three independent experiments run in triplicate; bars indicate SD. (*) indicate *p* ≤ 0.05 and (**) indicate *p* ≤ 0.01.

In the following set of experiments, we used melanoma spheroids, condition mimicking the 3D configuration of *in vivo* solid tumors [51]. The results showed effects comparable to those showed in the monolayer experiments (Figure 3A and 3B). However, the apoptotic effect of the Lansoprazole/Efavirenz (LE) combination was more marked than what we obtained with cell proliferation assays. Moreover, the combination of the two drugs led to an apoptotic response that was the sum of the two single treatments, suggesting a synergistic effect.

### Lansoprazole enhances Efavirenz antitumor efficacy in pulse administration protocol

**Figure 4:**
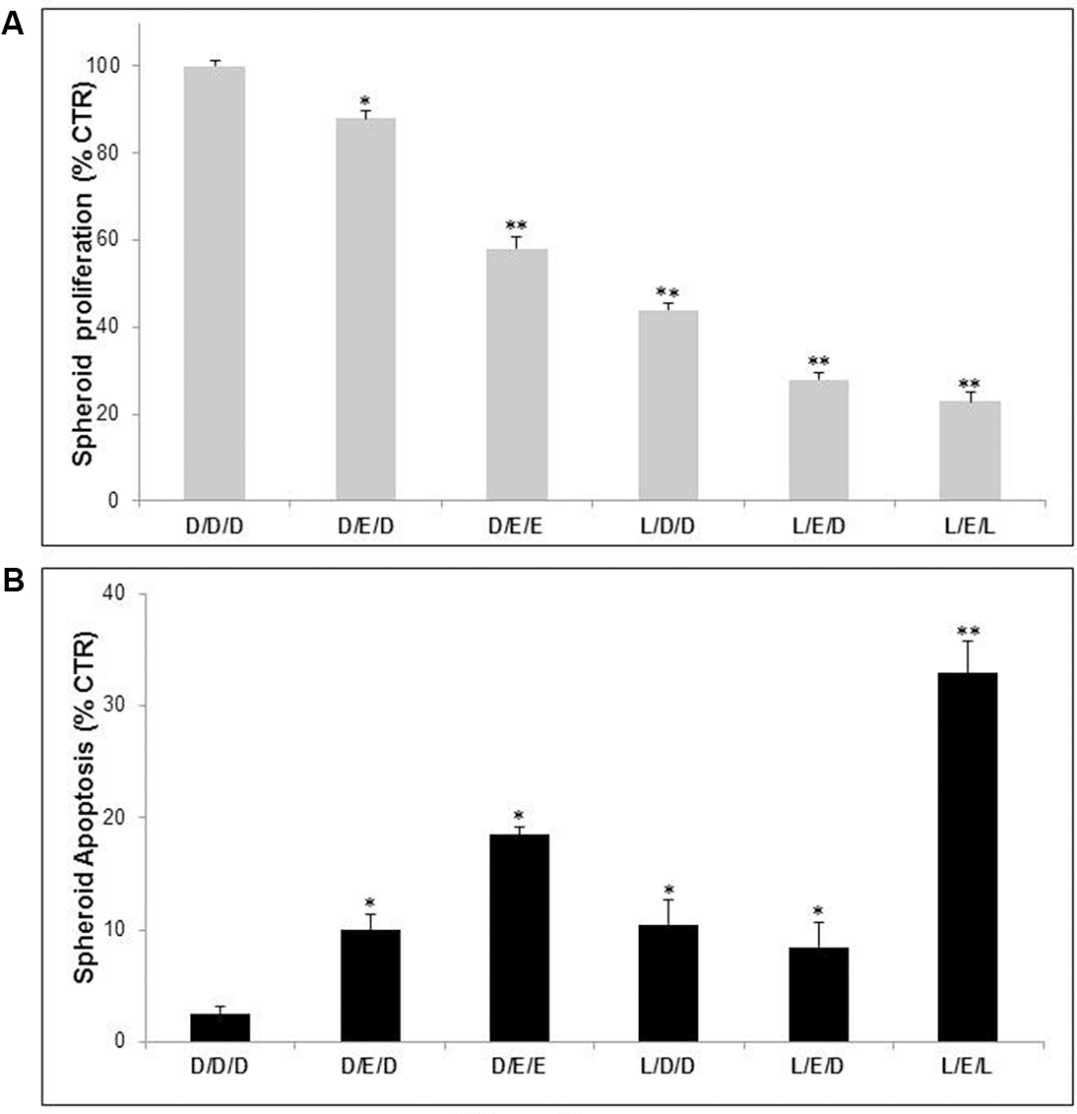
Lanzoprazole pre-treatment affected Efavirenz efficacy on 3D melanoma spheroids in pulse administration protocol Proliferative (**A**) and cytotoxic (**B**) effects of combined treatment of Lansoprazole (L) and EFV (E) on Me30966 melanoma spheroids. DMSO (D) D as control, L used for 48 hours (75 μM) and E for 96 hours (30 μM). Columns, mean percentages of cell death of three independent experiments run in triplicate; bars indicate SD. (*) indicate *p* ≤ 0.05 and (**) indicate *p* ≤ 0.01.

Clinically, in AIDS patients EFV discontinuing therapy is performed to reduce toxic effects, resulting in variable levels of unresponsiveness following a second round of therapy. The aim of this set of experiments was to evaluate whether LAN treatment performed during the window period between two EFV administrations could improve the antitumor efficacy of EFV. To this purpose melanoma spheroids were cultured in unbuffered condition with either single or combined treatments, as specified in Figure 4 legend. As showed in Figure 4 the most effective combination was that alternating LAN and EFV (LEL). This LAN/EFV combined treatment was more effective than two EFV administrations, using both proliferation and cytotoxicity assays, suggesting that LAN not only might be effectively used during the period of EFV interruption, but that it can well potentiate the effectiveness of EFV during the treatment periods. This was obtained with suboptimal drug concentrations, further suggesting that this approach may lead to a real reduction of the known EFV toxicity in all conditions where it is used as curative drug.

## DISCUSSION

Melanoma is a worldwide diffused tumor that still lacks an effective therapy for the advanced stages [52]. Progresses have been made through extensive screening programs aimed at an early tumor detection. Indeed, it has been ascertained that cutaneous melanoma can be cured if the neoplasm is surgically removed at an early stage, as defined in the Breslow classification [53]. When the tumor is detected at an advanced stage or if it is deeply developed passing the dermal layer, then there are few therapeutical options that can prevent or arrest the tumor progression [52]. Several protocols have been devised over the years, including the use of chemotherapy (either standard or limb perfusion), biological agents and immunotherapy drugs with limited success [54–59]. The recent use of monoclonal antibodies [60] is becoming to move something positive in this “pharmacological stalemate” in the treatment of metastatic melanoma. However, there is a urgent need of new more effective, less toxic and possibly less expensive therapeutical options. Here we provided evidence that the combination of proton pumps and reverse transcriptase inhibitors exerts a real effect against human malignant melanoma cells in both 2D and 3D culture conditions.

Previous studies of our group have shown that proton pump inhibitors have both a direct antitumor action [8, 37, 43, 44] and chemosensitizing activity [9, 33, 48, 49] against a variety of transplanted and spontaneous neoplasms in animals and humans. An ad hoc study has also shown that between the most representative molecules of this class of drugs, Lansoprazole has the most potent antitumor effect both *in vitro* and *in vivo* [8].

We have also shown that the reverse transcriptase inhibitor Efavirenz exerts a potent anti-tumor activity [3–5].

To date the unique clinical use of Efavirenz for the treatment of metastatic castration-resistant prostate cancer patients [20] is impaired by different side effects, due to the used higher dosage of Efavirenz respect to the 600 mg/day recommended for AIDS treatment. Lowering the daily dose of Efavirenz without affecting the drug's efficacy may be an efficient manner to control Efavirenz-related toxicity in some patients.

It is known that the tumor microenvironment is acidic [21, 27, 30–32] and that this peculiar tumor condition impairs the most of anti-tumor drugs effectiveness [9, 28, 33, 46–48]. Here we have shown that acidic pH medium remarkably reduced the antitumor effect of Efavirenz. The pre-treatment of melanoma cells with Lansoprazole (a prodrug that contrarily to the other drugs is activated in acidic condition) significantly improved the Efavirenz antitumor effect, supporting the results obtained with other therapeutic molecules [61]. The same results were obtained in 3D culture condition that reproduce the solid mass of tumors.

In this study we provided the proof of concept that Lansoprazole and Efavirenz can have a synergic action against human melanoma with a combined effectiveness almost equal to the sum of their single effects. More importantly, the alternate administration protocol of the two agents was the one showing the greatest effectiveness, thus providing the evidence that this sequential way of administration could be adopted in a clinical approach to cancer, together with decreasing the possible side effects associated with long term administration of Efavirenz. Furthermore, both agents have been used at suboptimal concentrations, thus opening an avenue for the adoption of more conservative protocols that while maintaining a significant efficiency at the same time will have a greater tolerability for the patients. Moreover, Efavirenz can be used in neoadjuvant therapies of melanoma and other malignant tumors in order to avoid tumor relapses following both surgery and chemotherapy. We also know that PPI can counteract the tumor immune escape due to the microenvironmental acidity [62, 63]. Finally, the results of this study give support to new investigations aimed at the setting up of either hybrids or combined molecules containing both Proton Pumps and Reverse Transcriptase inhibitors. In addition both Proton Pumps and Reverse Transcriptase may represent new targets for more specific and possibly less toxic anti-tumor molecules. However, these preclinical data obtained with commercially available drugs may hopefully trigger the setting up of clinical trials in order to translate these results to the patients’ bed. This is in part already occurred with PPI with clinical trials, four in human patients [46, 47, 50, 64] and two in domestic animals with spontaneous tumors [48, 49] and with EFV in a clinical trial performed in metastatic castration-resistant prostate cancer patients [20]. Of course we need data of a series of clinical trials obtained with either PPI or Efavirenz, in order to further support the use of Proton Pumps and RT-inhibitors combination therapies that this study is proposing.

## MATERIALS AND METHODS

### Chemicals and reagents

Lansoprazole was purchased by Sigma-Aldrich (Milan, Italy) and was dissolved at 20 mM in DMSO in absence of direct light and reconstituted immediately prior its use. Efavirenz was purified from commercially available Sustiva (Bristol-Myers Squibb) as described [3], dissolved in DMSO and added to the culture medium in the indicated concentrations and times. RPMI-1640 cell culture medium (BE12-702F), antibiotics (DE17-603E), phosphate buffer saline (PBS) (BE17-512F), trypsin/EDTA (BE17-171E) and fetal bovine serum (DE14-701F) were obtained from Lonza (Milan, Italy). Trypan blue was bought from Alexis Biochemicals (Florence, Italy) and Annexin V-FITC Apoptosis detection kit from Enzo Life Sciences (Lausen, Switzerland). 4-Nitrophenyl phosphate disodium salt hexahydrate tablets for proliferation assay was from Sigma-Aldrich.

The pH of all cell culture supernatants were estimated by the use of a pH 123 Microprocessor pH Meter (Hanna Instruments, Italy).

### Cells and spheroids

Human metastatic melanoma Me30966 (supplied by Istituto Nazionale per lo Studio e la Cura dei Tumori, Milan, Italy) was maintained in RPMI-1640 medium supplemented with 10% fetal calf serum and antibiotics, at 37°C in humidified 5% CO_2_. Experiments were performed in buffered (B), unbuffered (UNB) and pH6.5 RPMI-1640 cell culture medium (Lonza). The cell line was negative for mycoplasma contamination, as routinely tested by modified nested polymerase chain reaction (VenorGeM Kit, Minerva Biolabs, Germany).

To allow spheroid formation, 15 × 10^3^ Me30966 cells were cultured in 96-well plate (Costar Ultra Low Attachment, Sigma.Aldrich) in complete cell culture medium until 72 hours at 37°C and 5% CO_2_ in continuous rotation.

### Cell proliferation assay

Melanoma cells were plated at 1 × 10^4^ cells per well in 96-well plates in buffered RPMI medium (2D model) or at 15 × 10^3^ cells in 96-well plate in 0.2 ml cell culture medium (3D spheroid model). After 24 hours, the medium was replaced with fresh, unbuffered or acidic RPMI medium and cells were treated with EFV and/or Lansoprazole as specified in the results section. After treatment, cell proliferation was determined using 4-Nitrophenyl phosphate disodium salt hexahydrate tablets (Sigma-Aldrich) and the cell survival was evaluated by the 405 nm absorbance measured by a spectrophotometer ELx800 (Bio-Tek Instruments, Inc.). All experiments were run in triplicate wells and repeated at least twice.

### Cell death assay

Melanoma cells were plated at 3 × 10^5^ cells per well in 12-well plates in 1 ml of buffered RPMI medium (2D model), or at 15 × 10^3^ cells in 96-well plate in 0.2 ml medium (3D spheroid model). After 24 hours, the medium was replaced with unbuffered or acidic medium. After other 24 hours, necessary for cell medium adjustment, cells were treated with Lansoprazole e/o Efavirenz, following the reported experiments. Then, cells were collected by pooling cells from the medium (i.e., dead cells) and adherent (live) cells obtained by trypsinization. Cells were washed and resuspended in PBS with 0.4% trypan blue 1:1 (vol/vol) dilution or incubated with AnnexinV-FITC/Propidium Iodide for apoptosis detection (Enzo Life Sciences) as reported in the manufacturer's instruction. Cells were then analyzed by Flow cytometry on a Becton Dickinson FACScalibur using CellQuestPro software (Becton Dickinson System). For each sample the total events were acquired in 60 seconds. All experiments were run in triplicate wells and repeated at least twice.

### Statistical analysis

Data are presented as means ± SD with *n* = at least three independent sets of experiments and for triplicate wells/experiment. The statistical analysis was performed by Student's *t* test in all the reported experiments and the statistically significant differences were defined only when *p* < 0.05, using SigmaStat 4.0 software.

## Abbreviations

RT: endogenous non-telomerase reverse transcriptase
EFV: Efavirenz
LAN: Lansoprazole
